# Development of a Bead-Based Multiplex Assay for the Analysis of the Serological Response against the Six Pathogens HAV, HBV, HCV, CMV, *T. gondii*, and *H. pylori*

**DOI:** 10.3390/ht6040014

**Published:** 2017-10-30

**Authors:** Angela Filomena, Frank Pessler, Manas K. Akmatov, Gérard Krause, Darragh Duffy, Barbara Gärtner, Markus Gerhard, Matthew L. Albert, Thomas O. Joos, Nicole Schneiderhan-Marra

**Affiliations:** 1 NMI Natural and Medical Sciences Institute at the University of Tuebingen, 72770 Reutlingen, Germany; angela.filomena@gmx.de (A.F.); thomas.joos@nmi.de (T.O.J.); 2 TWINCORE, Centre for Experimental and Clinical Infection Research, 30625 Hanover, Germany; pessler.frank@mh-hannover.de (F.P.); manas.akmatov@helmholtz-hzi.de (M.K.A.); gerard.krause@helmholtz-hzi.de (G.K.); 3 Helmholtz Centre for Infection Research, 38124 Brunswick, Germany; 4 Centre for Individualized Infection Medicine, 30625 Hanover, Germany; 5 Translational Infrastructure Epidemiology, German Centre for Infection Research (DZIF), 30625 Hanover, Germany; 6 Immunobiology of Dendritic Cells, Institute Pasteur, 75015 Paris, France; darragh.duffy@pasteur.fr (D.D.); matthew.albert@pasteur.fr (M.L.A.); 7 Inserm U1223, Institute Pasteur, 75015 Paris, France; 8 Centre for Translational Research, Institute Pasteur, 75015 Paris, France; 9 Institut für Mikrobiologie und Hygiene, 66421 Homburg/Saar, Germany; barbara.gaertner@uks.eu; 10 TUM Technical University of Munich, 80333 Munich, Germany; markus.gerhard@tum.de

**Keywords:** multiplex, serotest, multi-pathogen assay, seroprevalence, hepatitis, cytomegalovirus (CMV), *Toxoplasma gondii*, *Helicobacter pylori*

## Abstract

The spread of infectious diseases and vaccination history are common subjects of epidemiological and immunological research studies. Multiplexed serological assays are useful tools for assessing both current and previous infections as well as vaccination efficacy. We developed a serological multi-pathogen assay for hepatitis A, B and C virus, cytomegalovirus (CMV), *Toxoplasma gondii*, and *Helicobacter pylori* using a bead-based multiplex assay format. The multi-pathogen assay consisting of 15 antigens was utilized for the analysis of the serological response in elderly individuals of an influenza vaccination study (*n* = 34). The technical assay validation revealed a mean intra-assay precision of coefficient of variation (CV) = 3.2 ± 1.5% and a mean inter-assay precision of CV = 8.2 ± 5.3% across all 15 antigens and all tested samples, indicating a robust test system. Furthermore, the assay shows high sensitivities (ranging between 94% and 100%) and specificities (ranging between 93% and 100%) for the different pathogens. The highest seroprevalence rates in our cohort were observed for hepatitis A virus (HAV; 73.5%), followed by CMV (70.6%), *T. gondii* (67.6%) and *H. pylori* (32.4%). Seroprevalences for hepatitis B virus (HBV, 8.8%) and hepatitis C virus (HCV, 0%) were low. The seroprevalences observed in our study were similar to those from other population-based studies in Germany. In summary, we conclude that our multiplex serological assay represents a suitable tool for epidemiological studies.

## 1. Introduction

The spread of infectious diseases and vaccination history are common subjects of epidemiological and immunological research studies. Chronic or latent pathogen infections can be a strong risk factor for several pathologies such as cancer [1,2], rheumatic diseases [3,4] and other chronic illnesses [5,6]. Furthermore, infections of pregnant women, with for example the TORCH pathogens (*Toxoplasma gondii*, syphilis, varicella-zoster, parvovirus B19, Rubella, CMV, and Herpes), are known to cause severe outcomes to their fetus and new born infants [7]. 

Commercially available screening assays, e.g., enzyme-linked immunosorbent assays (ELISA), can test only for antibodies against one pathogen in a sample. Therefore, a multitude of different ELISAs have to be used to perform epidemiological studies on seroprevalences of different pathogens, which is associated with higher costs and logistics. In contrast, multiplex technologies allow the analysis of different parameters in one sample. Thus, a multiplexed serological assay may be a useful tool for assessing both current and previous infections as well as vaccination efficacy and/or history. The Luminex xMAP technology (Luminex Corp., Austin, TX, USA) offers the possibility to differentiate up to 500 different color-coded paramagnetic bead populations, which can be measured by the FLEXMAP 3D analyzer (Luminex Corp.). By immobilizing one antigen on a certain bead population and mixing different bead populations after antigen immobilization, multiplex serology becomes possible. It has been shown before that antibodies towards multiple pathogens can be detected simultaneously in a multiplex assay setup using the Luminex xMAP technology [8,9].

Here, we present the development and validation of a bead-based multi-pathogen serological assay for hepatitis A virus (HAV), hepatitis B virus (HBV), hepatitis C virus (HCV), CMV, *T. gondii*, and *Helicobacter pylori*. The assay was initially developed for HCV not only to differentiate negative and positive antibody response but also to discriminate acute from chronic HCV infection. Due to the fact that other pathogens, e.g., HAV, HBV, CMV, *T. gondii*, and *H. pylori*, can cause the same more or less unspecific symptoms as an acute HCV infection, the assay was expanded to these other pathogens. We used the developed multi-pathogen assay to assess the seroprevalences of the above mentioned infections as well as HAV and HBV vaccination history in an influenza vaccination study cohort.

## 2. Materials and Methods 

### 2.1. Antigens

In total, 15 antigens deriving from six different pathogens (HAV, HBV, HCV, CMV, *T. gondii*, and *H. pylori*) were used in the serological bead-based multi-pathogen assay. For HAV four antigens were used: a formalin inactivated full virus preparation and three purified structural proteins (VP4-VP2, VP3, and VP1) recombinantly expressed in *Escherichia coli*. For HBV, three antigens were used: purified hepatitis B core antigen (HBcAg) recombinantly expressed in *E. coli* and two hepatitis B surface antigen (HBsAg) preparations (HBsAg ad and HBsAg ay) highly purified from human plasma. For HCV five antigens were used: four purified proteins (Core g4a, Core g1b, NS3 g1a, and NS3 g1b) recombinantly expressed in *E. coli* and a customized peptide (c22 g1a). For CMV, *T. gondii*, and *H. pylori*, one antigen was used for each pathogen: for CMV, a whole cell lysate of strain AD169, for *T. gondii*, a sonicate of whole tachyzoites (RH strain), and for *H. pylori*, a soluble protein extract of strain 49503.

### 2.2. Human Sera

For the development of the serological bead-based multi-pathogen assay, a variety of samples positive and negative for the six pathogens was necessary. Fifteen samples negative for anti-HAV IgG, anti-HBcAg IgG, and anti-HBsAg IgG, 15 samples positive for anti-HBsAg IgG, and anti-HAV IgG, 15 samples positive for anti-HBcAg IgG and anti-HBsAg IgG, 10 samples negative for anti-CMV IgG, 20 samples positive for anti-CMV IgG, 10 samples negative for anti-Toxo IgG, and 10 samples positive for anti-Toxo IgG were purchased from Biomex (Heidelberg, Germany). Anti-HAV IgG were determined using the ARCHITECT i1000SR Anti-HAV Ab IgG Reagent Kit (Abbott Laboratories, Abbott Park, IL, USA), anti-HBcAg IgG by ARCHITECT i1000SR Anti-HBc II Ab IgG Reagent Kit (Abbott Laboratories, Abbott Park, IL, USA), anti-HBsAg IgG by ARCHITECT i1000SR Anti-HBs Ab IgG Reagent Kit (Abbott Laboratories), anti-CMV IgG by ARCHITECT i1000SR Anti-CMV IgG Reagent Kit (Abbott Laboratories) and anti-Toxo IgG by ARCHITECT i1000SR Anti-Toxo IgG Reagent Kit (Abbott Laboratories).

Samples positive (*n* = 141) and negative (*n* = 87) for HCV were received during the European Commission FP7 project SPHINX. HCV was identified by positive HCV-RNA (reverse-transcriptase polymerase chain reaction; RT-PCR). Protocols were reviewed and approved by local ethical committees and conform to the ethical guidelines of the 1975 Declaration of Helsinki. The healthy control sera confirmed to be negative for HCV were obtained from blood banks in Cairo (*n* = 26) and from the Etablissement Français du Sang (French National Blood Service) France (*n* = 61). Another 30 samples negative for HCV antibodies were purchased from Seralab (Sera Laboratories International Ltd., West Sussex, United Kingdom). Furthermore, 20 samples of patients acutely infected with HAV (positive anti-HAV IgM Ab) and another 20 samples of patients acutely infected with HBV (positive by Corzyme M; ribosomal DNA; Abbott Laboratories (Abbott Park, IL, USA) and Auszyme Monoclonal; third-generation enzyme immunoassay; Abbott Laboratories) were also obtained during the SPHINX project. The samples are described elsewhere [10]. In addition, a set of samples positive (*n* = 92) or negative (*n* = 142) for anti-HBc IgG (ARCHITECT i1000SR Anti-HBc II Ab IgG Reagent Kit, Abbott Laboratories) was obtained from Barbara Gärtner (Institut für Mikrobiologie und Hygiene, Homburg/Saar) and fall under the central ethical statement 2003. The Technical University of Munich (TUM) provided samples positive (*n* = 414) and negative (*n* = 338) for *H. pylori*. The diagnosis as positive or negative for a *H. pylori* infection was determined by Warthin–Starry silver stain. The study was approved by the ethics committee of the TUM (20 May 2009, No. 2453/09). The study cohort is described elsewhere [11].

The developed multi-pathogen assay was used to analyze the serological response in a pilot influenza vaccination study cohort consisting of 162 samples from a total number of 34 study participants aged 65–80 years (median 70 years) recruited from the general population. The study cohort is described elsewhere [12]. The samples of study day 0 were screened with a commercial CMV ELISA (CMV-IgG-ELISA PKS, medac GmbH, Wedel, Germany). The study was carried out with the approval of the ethics committee of Hanover Medical School (file no. 6775) and in accordance with national law and the Helsinki Declaration (in its current, revised form: 64th WMA General Assembly, Fortaleza, Brazil, October 2013). Written informed consent was obtained from all study participants before study assessments were begun. The study was registered on ClinicalTrials.gov database (no. NCT02362919) prior to starting. 

### 2.3. Antigen Immobilization

The immobilization of proteins was performed as described previously [13]. Briefly, a magnetic particle processor (KingFisher 96, Thermo Scientific, Schwerte, Germany) was used to immobilize the proteins on paramagnetic carboxylated beads (MagPlex microspheres, Luminex Corp., Austin, TX, USA). The proteins were covalently immobilized onto the beads using 1-Ethyl-3-(3-dimethylaminopropyl)carbodiimide (EDC)/sulfo-N-hydroxysuccinimide (sulfo-NHS) chemistry and a coupling concentration of 50 μg/mL per protein. After immobilization, beads were stored at 4°C until further use.

The HCV peptide c22 g1a was immobilized to a carrier protein using an amine-to-sulfhydryl crosslinker. First, the carrier protein bovine serum albumin (BSA; coupling concentration: 100 μg/mL) was immobilized on beads using EDC/sulfo-NHS chemistry as described previously [13]. These BSA-beads were activated for 1 h at room temperature (RT) with 100 μL sulfosuccinimidyl 4-(N-maleimidophenyl)butyrate (sulfo-SMPB) solution (1.5 mg/mL) + 100 μL PBS + 0.01% (*v*/*v*) Triton X-100. The cysteine containing peptide was reduced with one molar equivalent tris(2-carboxyethyl)phosphine (TCEP). Therefore, 50 μL cysteine peptide solution (1 mM) was incubated with 50 μL TCEP solution (1 mM in PBS) for 20 min at RT. At the end of the reaction, 150 μL PBS + 0.0125% (*v*/*v*) Triton X-100 was added. Activated beads were washed twice with 500 μL PBS + 0.005% (*v*/*v*) Triton X-100. The reduced peptide was incubated with the activated beads for 1 h at room temperature (RT). Beads were washed twice with 500 μL PBS + 0.005% (*v*/*v*) Triton X-100, resuspended in 300 μL CBS (PBS + 1% (*w*/*v*) BSA) + 0.1% ProClin and stored at 4 °C until further use.

The HAV formalin inactivated full virus preparation was immobilized according to the manufacturers’ protocol with Mix & Go from Anteo Diagnostics (AMG Activation Kit for Multiplex Microspheres) using coordination chemistry.

### 2.4. Bead-Based Multiplex Assay Procedure

All single-bead populations with immobilized antigens were combined for usage in the multiplex assay procedure. Additionally, four internal control beads were added: (1) beads with covalently immobilized human IgG as control for the detection system, (2) beads with goat-anti-human IgG antibody as control for the sample addition, and (3 + 4) beads with BSA and beads with *E. coli* lysate as control for unspecific binding.

Plasma samples were prediluted 1:40 in PVXC (PBS + 0.8% polyvinylpyrrolidone (PVP) + 0.5% polyvinyl alcohol (PVA) + 0.1% casein) followed by a 1:5 dilution in sample buffer (50% (*v*/*v*) CBS + 50% (*v*/*v*) LowCross-Buffer (Candor Bioscience GmbH, Wangen, Germany) + 1 mg/mL *E. coli* lysate + 0.5 mg/mL purified glutathione *S*-transferase (GST)), yielding a final 1:200 dilution of the samples. After dilution, the samples were incubated for 20 min at RT. The bead mixture was diluted in assay buffer (CBS + 0.05% Tween 20) containing approximately 20 beads/μL per single-bead population and distributed on a 96 well PCR plate (50 μL per well). The assay was performed in a semi-automated fashion using the magnetic particle processor mentioned above. The beads were transferred from the bead source plate to 50 μL of the diluted human plasma samples and incubated for 2 h at RT. Unbound antibodies were removed by washing the beads twice with wash buffer (100 μL PBS + 0.05% Tween 20). To detect bound human IgGs the beads were incubated for 1 h at RT with 50 μL of an R-phycoerythrin (R-PE) labeled goat-anti-human IgG antibody (Jackson ImmunoResearch, West Grove, PA, USA) diluted to 5 μg/mL in assay buffer. After two washing steps with 100 μL wash buffer the beads were resuspended in 100 μL wash buffer. Measurements were performed using a Luminex FLEXMAP 3D instrument operated with Luminex xPONENT software version 4.0 (Luminex Corp.). Binding events were displayed as median fluorescence intensities (MFI) based on ≥50 measured beads per bead population. Every sample had its own sample specific background. The sample specific background was the mean of the MFI values on the BSA bead and the *E. coli* lysate bead, which was subtracted from the MFI values on the antigen carrying beads of the corresponding sample. 

### 2.5. Technical Assay Validation

The multi-pathogen assay was technically validated with respect to intra- and inter-assay precision. Assay precision is indicated as the percentage coefficient of variation (% CV). For determination of the intra-assay precision 20 replicates of a sample were measured on one assay plate. For calculation of the inter-assay precision, triplicates of a sample were measured on four experimental days. The determination of the precision was performed for the blank, a negative sample, and four positive samples. 

### 2.6. Cutoff Definition and Methods for Sample Classification

A cutoff was calculated for every antigen based on the reactivity in negative control sera. In this study 15 negative sera for HAV and HBV were used, for HCV 28, for CMV and *T. gondii* each 10 and for *H. pylori* 50 negative sera. Outliers were identified using the interquartile range (IQR). Values were considered to be outliers when the value was below Q1 – 1.5 × IQR or above Q3 + 1.5 × IQR. Outliers were excluded from the cutoff calculations. The cutoff value for every antigen was defined as the mean MFI value + 3× standard deviation. Dividing the antigen-specific MFI value of the samples by the antigen-specific cutoff yielded the signal-to-cutoff (S/CO) values.

To classify a sample into negative, borderline or positive for HAV, HBV, CMV, *T. gondii*, or *H. pylori* the S/CO value of one pathogen-specific antigen was considered: positive samples have a S/CO > 2, negative samples have a S/CO < 1 and borderline samples have a S/CO value between 1 and 2. For HAV, the antigen formalin inactivated full virus preparation was used for classification, for HBV, the antigen HBcAg, and for CMV, *T. gondii,* and *H. pylori*, the lysate of each pathogen was used. To classify a sample as negative, borderline, or positive for HCV, the S/CO value of all five antigens (Core g4a, Core g1b, c22 g1a, NS3 g1a and NS3 g1b) was considered. In addition, HCV-positive samples were classified into acute or chronic infection (method development not published so far). The criteria for HCV status classification is shown in Table 1.

Furthermore, HAV-positive samples could be distinguished into positive by vaccination or by infection. Therefore, the reactivity of the antigens VP4-VP2, VP3, and VP1 was considered: samples from patients with HAV infection have a S/CO > 1 on all three antigens, while samples from vaccinated patients have two or less antigens with a S/CO > 1.

Also for HBV, a more detailed classification of the samples was possible. A HBV positive sample could be further classified as “cleared” or “non-cleared.” Therefore, the reactivity of the antigens HBsAg ad and HBsAg ay was considered: positive samples from cleared patients have at least one HBsAg with a S/CO > 1. HBV-positive samples without any reactivity against HBsAg (S/CO < 1) are considered to be non-cleared. Moreover, it was determined if HBV-negative study participants have received a vaccination against HBV. Negative study participants being vaccinated against HBV have at least one HBsAg with a S/CO > 1.

### 2.7. Statistical Analysis

To evaluate the significance of differences between two distributions, the nonparametric Mann–Whitney *U* test was used (*p*-values). Receiver operating characteristic (ROC) curve analysis was used to illustrate the diagnostic ability of an antigen. In a first step of the method development for the classification of samples, a random forest algorithm was used to get an impression of the diagnostic ability of the assay. *p*-Values, ROC curve analysis, and sample classification with a random forest algorithm were used for antigen evaluation. Statistical analyses were performed using R (RStudio V 0.97, Boston, MA, USA) and WEKA V 3.6 (The University of Waikato, Hamilton, New Zealand).

## 3. Results

### 3.1. Assay Development and Performance 

For the development of the bead-based multi-pathogen assay, a primary set of 64 pathogen-specific antigens including different variants of antigens were immobilized on different beads. Plasma or serum samples from positive and negative donors for HAV, HBV, HCV, CMV, *T. gondii*, and *H. pylori* were measured with the primary multi-pathogen assay. The performance of every antigen was evaluated based on the height of the background signal and the diagnostic ability using the following approaches, like *p*-values, ROC curve analysis, and sample classification with a random forest algorithm (data not shown). Only the best-performing antigens were included in the final setup of the multi-pathogen assay consisting of 15 antigens from the six different pathogens. 

The technical validation of the 15-plex multi-pathogen assay revealed a mean intra-assay precision of CV = 3.2 ± 1.5% and a mean inter-assay precision of CV = 8.2 ± 5.3% across all 15 antigens and all samples. 

The diagnostic ability for every pathogen in the multi-pathogen assay was assessed on the basis of the classification results of samples from positive and negative donors for HAV, HBV, HCV, CMV, *T. gondii*, and *H. pylori*. The classification results with the corresponding diagnostic quality criteria are shown in Table 2. The overall diagnostic accuracy was very high for all pathogens and varied between 93.8% and 100%. For HBV, HCV, and *H. pylori*, a larger second sample set was also available for the validation of the diagnostic ability (Table 3). 

HAV and HBV infections are vaccine-preventable diseases. Thus, another aim of the multi-pathogen assay was to assess if the blood donors were vaccinated for HAV and/or HBV. A positive HAV test result is caused by vaccination or by natural infection. For further classification of HAV positive samples the reactivity against the antigens VP4-VP2, VP3, and VP1 was considered. It could be observed that only samples from patients with an HAV infection had a reactivity (S/CO > 1) on all these three single antigens (Table 4). 

The vaccine for HBV contains HBsAg, thus samples from vaccinated donors show an antibody reactivity against HBsAg, but no antibody reactivity against HBcAg. In the multi-pathogen assay two variants of the HBsAg were used, one from serotype ad and the other from serotype ay. Samples from donors with and without vaccination against HBV were analyzed with the multi-pathogen assay and results for the HBsAg variants were compared (Table 5). Whereas HBsAg ad shows a higher sensitivity, HBsAg ay possesses a higher specificity.

### 3.2. Analysis of an Influenza Vaccination Study

The multi-pathogen assay was used to screen samples from an influenza vaccination study consisting of 34 participants. For every participant, there was a sample before and several samples after receiving the influenza vaccine Fluad. The screening results with the multi-pathogen assay were analyzed for every sample. Samples were classified as positive, negative or borderline for the six pathogens, HAV positive samples were further classified as positive due to vaccination or natural infection, and HBV negative samples were checked for HBV vaccination. It has to be noted that antibodies against HBsAg can also occur in samples of donors with antibody reactivity against HBcAg. If this is the case, it is very likely that a former infection with HBV has been cleared by the immune system. Therefore, samples, which have been classified as HBV positive, were further classified in “cleared” (positive with anti-HBs) or “non-cleared” (positive without anti-HBs). Also, for HCV-positive samples, a further classification of the infection status into “acute” or “chronic” was conducted. Having more than one sample per participant made it complicated to have a consistent classification for a certain pathogen. If the samples from the different time points yielded unequal classification results, a participant was classified as “ambiguous” for the corresponding pathogen. Results of the pilot influenza vaccination study are shown in Table 6. 

Positive test results were most commonly received for HAV (73.5%), followed by CMV (70.6%), *T. gondii* (67.6%), and *H. pylori* (32.4%). Nearly all HAV-positive patients seemed to be positive due to vaccination (84%). Four study participants did not show consistent classification results for HAV, and three of them showed S/CO values around 1, leading to a negative or borderline classification. The fourth ambiguous HAV study participant showed mostly borderline classification but was once classified as positive (S/CO = 2.2). 

Only three study participants were classified as positive for HBV. Two of the three HBV positive study participant showed S/CO values < 5 for HBcAg, but the third showed S/CO values of around 46. This third participant did also possess high S/CO values for HBsAg ad (~840) and HBsAg ay (~36). One of the other two positive HBV study participants did not show any antibody reactivity against HBsAg and the other patient had S/CO values for HBsAg ay of around 3, but no reactivity against HBsAg ad. Therefore, the two study participants, which were anti-HBs positive, were classified as cleared, whereas the other one was non-cleared. However, two study participants were ambiguous because the samples were classified as negative or borderline (S/CO values ~1) for HBV. Altogether, most of the participants were negative for HBV, but 41.4% of them showed a HBV vaccination pattern.

For HCV, most of the study participants were negative (82.4%), no one was tested positive, and six participants were classified as ambiguous. Four participants were ambiguous because the samples were classified as negative or borderline, but two participants showed some positive samples (Table 7). Especially on the antigen NS3 g1a there were high S/CO values in several samples. The first study participant was classified as negative on day 0 and 3, but positive on day 7, 21, and 70, with a peak of reactivity on day 21. Aside from HCV, this participant was tested positive for HAV and CMV, but the reactivity on these antigens remained very constant (CV = 3.7% for HAV and CV = 9.7% for CMV). The second study participant was classified as positive on day 0, 3, 7, and 21, but borderline on day 70. With highest reactivity on day 3, a clear decline of reactivity could be seen until day 70. In addition, this participant was tested positive for HAV as well as *T. gondii*, and the reactivity on these antigens also remained very constant (CV = 3.2% for HAV and CV = 13.1% for *T. gondii*), as it was observed for the first study participant.

**Figure 1:**
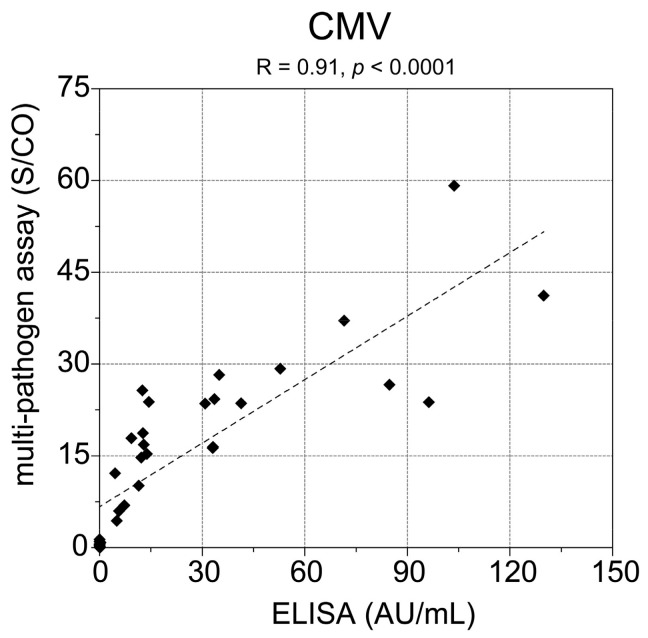
Correlation of the cytomegalovirus (CMV) results from the multi-pathogen assay and enzyme-linked immunosorbent assays (ELISA) measurement. Samples from day 0 of the study were used for comparison. Spearman’s ρ is 0.91 with *p* < 0.0001.

Besides the high positivity rate for CMV, three study participants could not be classified unambiguously for CMV. The samples of these participants were classified as negative or borderline with S/CO values of 1.24 ± 0.25, 1.15 ± 0.29 and 1.2 ± 0.6. The samples of study day 0 (first visit of the participants) were also screened with a commercial CMV ELISA (CMV-IgG-ELISA PKS, medac GmbH, Wedel, Germany) and the comparison with the results of our multi-pathogen assay are shown in Figure 1. A high correlation of measurement results of the two methods was found (Spearman’s ρ = 0.91, *p* < 0.0001). The same samples were classified as positive with both assays.

However, the classification for *T. gondii* of one participant was ambiguous due to some samples classified as positive and some as borderline (S/CO = 2.46 ± 0.57). Furthermore, three study participants were ambiguous for *H. pylori* with S/CO values of 0.85 ± 0.49, 1.03 ± 0.19 and 4.23 ± 2.69. 

**Figure 2:**
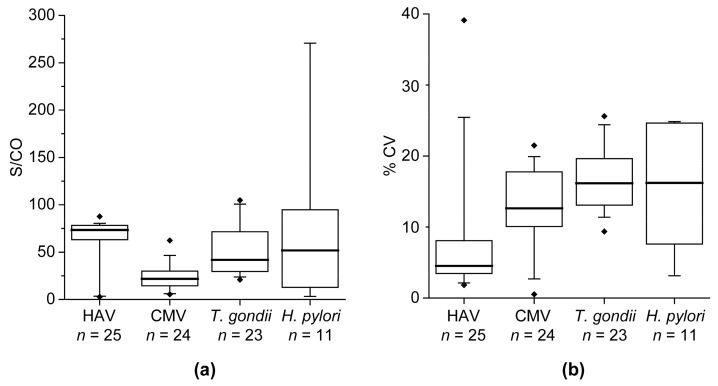
Boxplots of S/CO values and % CV in study participants positive for HAV, CMV, *T. gondii*, or *H. pylori*. For every study participant, 2–5 samples from different time points after influenza vaccination were available. The mean S/CO of every participant is shown in (**a**) and the corresponding calculated CV values of the participants are illustrated in (**b**).

In general, it was observed that even if S/CO values vary between different study participants, the variance within the samples of one participant (% CV) is relatively low (Figure 2). As illustrated in Figure 2a, the variance of S/CO values for HAV and CMV is lower than for *T. gondii* and *H. pylori*. However, lowest CV values were received for HAV (mean = 8.0, standard deviation (SD) = 8.5). Even if CV values for CMV (mean = 12.7, SD = 5.3), *T. gondii* (mean = 16.6, SD = 4.4), and *H. pylori* (mean = 15.3, SD = 8.2) were higher, 85.5% of the study participants showed CV values <20% (Figure 2b).

## 4. Discussion

Here, we present a serological bead-based immunoassay for the simultaneous detection of antibodies against six different pathogens: HAV, HBV, HCV, CMV, *T. gondii*, and *H. pylori*. CV values obtained during technical assay validation (intra-assay CV of 3.2 ± 1.5% and inter-assay CV of 8.2 ± 5.3%) were below 15%, which is a commonly accepted range for research assays and indicate a robust test system. Positive and negative samples for every pathogen were used to evaluate the diagnostic ability. Overall, the multi-pathogen assay showed a very good performance in an initial set of samples with sensitivities of 94–100% and specificities of 93–100%. Comparable results to the initial set of samples were also achieved with the validation set of samples for HBV and HCV. Only the specificity of the *H. pylori* antigen was in the validation set lower (85.9%) than in the initial set of samples (97.5%). The sensitivity was still very high (96.8%). In the validation set of samples, the clinical classification as positive or negative for a *H. pylori* infection was determined by Warthin–Starry silver stain and not by an antibody testing against *H. pylori*. Therefore, a lower value for the specificity was expected because the serological antibody measurement cannot differentiate past from present infections. We assume that some of the patients falsely classified as positive for *H. pylori* have had an infection and cleared the bacteria, so that they were no more positive by Warthin–Starry silver stain. However, even if the specificity for *H. pylori* was lower in the validation set than in the initial set of samples, the diagnostic ability is still higher compared to Michel et al. [14], who received 89% sensitivity and 82% specificity using a multiplex *H. pylori* serological assay.

However, overall our assay revealed very high concordance (>95%) with the clinical classification of the tested samples, which is much higher in comparison to another protein microarray for simultaneous detection of antibodies against five human hepatitis viruses by Xu et al. [15], who received >85% concordance. Furthermore, Augustine et al. [9] also developed a multiplex immunoassay for different environmental pathogens including HAV, *T. gondii* and *H. pylori* and they received 89%, 100% and 87.5% accuracy respectively. We observed the same accuracy for *T. gondii* and a higher accuracy for HAV (98%) and *H. pylori* (92.5%).

Recently, Ye et al. [16] presented a method to discriminate vaccine-induced immunity from natural infection with HAV. They developed an ELISA based on a recombinant protein containing six immune-dominant epitopes from the nonstructural HAV proteins. In contrast, our method used three structural HAV proteins and a formalin inactivated full virus preparation. We observed that all samples (100%) from donors having received an HAV vaccination and 19 of 20 samples (95%) from patients acutely infected with HAV could be classified correctly with our method. In comparison, Ye et al. received a sensitivity of 93.8% and a specificity of 91.0%.

Although the HBV and HCV validation samples could verify the diagnostic ability received in the initial set of samples, in the future also validation samples for HAV, CMV, and *T. gondii* have to be analyzed with the multi-pathogen assay. In addition, studies involving bigger samples sizes of individuals vaccinated against HAV and HBV are necessary for verification of the classification criteria used in this study. In general, often better values for sensitivity and specificity can be achieved by measuring a higher number of samples, whereby few falsely classified samples are of less consequence in calculated percentages. However, this is one difficulty in the clinical validation of multiplex analysis: a suitable number of samples for all represented pathogen detections is required with an appropriate reference classification test result. In fact, we also used antigens from hepatitis D virus (HDV), hepatitis E virus (HEV), and Epstein–Barr virus (EBV), but did not yet have the appropriate classified samples (positive and negative) to validate the assay for these viruses. Therefore, we could not set a cutoff, calculate the diagnostic ability of the antigens, or use them for analysis of the serological response in the study cohort.

The analysis of the serological response in the influenza vaccination study showed positive test results most commonly for HAV (73.5%), which is in accordance with the seroprevalence (63.8–83.4%) among elderly individuals in Germany [17]. In contrast to this, we received an HAV vaccination coverage of 61.8%, which is higher than 11.9–20.6% reported by Poethko-Müller and Schmitz [18]. Also, the HBV vaccination coverage was higher in our study (41.8%) than the 9.5–16.4% reported by Poethko-Müller and Schmitz [18]. However, the analyzed cohort here was very small (34 study participants), and due to the fact that it was an influenza vaccination study cohort, the individuals could have a higher likelihood to receive vaccinations.

With three study participants tested positive for anti-HBc (8.8%), we observed an only slightly higher prevalence for HBV than the reported prevalence in Germany of 5.1% [17]. Two of the three anti-HBc positive study participants were also positive for anti-HBs and therefore classified as cleared. The other one was anti-HBs negative and classified as non-cleared, but due to very low S/CO values on HBcAg it could be possible that HBV clearance occurred a very long time ago, so that the anti-HBs titer declined below the detection limit of the assay. 

In our study, no participant was unambiguously classified as HCV-positive, which conforms to the very low prevalence of 0.3% in Germany [17]. Nevertheless, two participants showed some positive longitudinal samples for HCV with first an increase and then a decrease of S/CO values on the antigen NS3 g1a, whereas reactivity against antigens of other pathogens remained very constant. This observation might be caused by cross-reactivity induced by the influenza vaccination. Based on the HCV reactivity profile also an acute infection with HCV followed by a clearance of the virus is possible, but due to low prevalence of HCV in Germany this explanation is not very likely.

Besides high HAV seropositivity, we also observed a high number of seropositive study participants for CMV (70.6%), which is in accordance with the seroprevalence >70% in German individuals aged >60 years as reported by Lübeck et al. [19]. Other studies often investigate younger age groups, explaining why lower seroprevalence is observed, e.g., 27% in children aged 1–17 years [20], 42% in donors aged 20–40 years [21], or 42% in females aged <45 years [22]. In addition, the comparison of the multi-pathogen assay with a commercial CMV ELISA showed 100% concordance of the samples being tested positive for CMV. However, our observed result for the seroprevalence of *T. gondii* at 67.6% is comparable to other studies reporting >55% in donors older than 45 years [23] or 77% in German adults aged 70–79 years [24]. Furthermore, 32.4% of the participants in our study cohort showed antibodies against *H. pylori*, which is in accordance with previous investigations with 28.9 % [25] or ~40% seropositivity [26] in comparable age groups.

Even if signals on the antigens varied between the different participants, the variance within the longitudinal samples of one participant was relatively low. Nevertheless, some study participants could not be classified unambiguously for some pathogens because S/CO values of the corresponding antigen fluctuated around the cutoff. In these cases, the low variance can still cause a misclassification of the samples. However, antibody titers in tested persons are not always static and can change over time.

In total, the presented serological bead-based immunoassay for the simultaneous detection of antibodies against six different pathogens is robust, sensitive, specific, and a suitable tool for epidemiological studies. It can serve to simultaneously investigate seroprevalences for several pathogens. By screening samples of a patient from different time points, changes in the seropositivity against different pathogens can be monitored, e.g., before and after vaccination or infection. The integration of antigens from other pathogens would make another application of the multi-pathogen serological assay possible, e.g., screening of pregnant women for antibodies against the TORCH pathogens, as it is known that infections with these pathogens during pregnancy can cause severe outcomes to their fetus and newborn infants. However, one of the major challenging parts in the development of a multi-pathogen serology test is the availability of respective validation samples.

## Figures and Tables

**Table 1:** Criteria for hepatitis C virus (HCV) status classification.

HCV Status	Criteria
negative	neither borderline nor positive
borderline	min. 3 antigens * with a S/CO value between 1 and 2 or exactly 2 antigens * with a S/CO value between 1 and 2 and simultaneously the sum of the S/CO from NS3 g1a and NS3 g1b is ≥ 3.2
positive	min. 3 antigens* with a S/CO > 2 or 2 antigens * with a S/CO > 2 and simultaneously the sum of the S/CO from NS3 g1a and NS3 g1b is ≥ 3.2
positive with acute infection	sum of the S/CO values of HCV antigens * < 5808 or sum of the S/CO values of HCV antigens * > 5808 in combination with an alanine aminotransferase (ALT) activity > 500 IU/L.
positive with chronic infection	sum of the S/CO values of HCV antigens * > 5808 and an ALT activity < 500 IU/L

* Core g4a, Core g1b, c22 g1a, NS3 g1a and NS3 g1b. S/CO: signal-to-cutoff.

**Table 2:** Diagnostic ability of the developed multi-pathogen assay to identify selected pathogens in an initial set of samples.

		HAV	HBV	HCV	CMV	*T. gondii*	*H. pylori*
classification	true positive	34	33	33	19	10	36
	false positive	0	2	0	0	0	1
	false negative	1	2	0	1	0	1
	true negative	15	28	26	10	10	39
diagnostic accuracy (%)		98.0	93.8	100	96.7	100	97.4
sensitivity (%)		97.1	94.3	100	95.0	100	97.3
specificity (%)		100	93.3	100	100	100	97.5
positive predictive value (%)		100	94.3	100	100	100	97.3
negative predictive value (%)		93.8	93.3	100	90.9	100	97.5

HAV: hepatitis A virus; HBV: hepatitis B virus; HCV: hepatitis C virus; CMV: cytomegalovirus.

**Table 3:** Diagnostic ability of the developed multi-pathogen assay to identify selected pathogens in a validation set of samples.

		HBV	HCV	*H. pylori*
classification	true positive	86	108	365
	false positive	3	1	35
	false negative	6	0	12
	true negative	139	90	213
diagnostic accuracy (%)		96.2	99.5	92.5
sensitivity (%)		93.5	100	96.8
specificity (%)		97.9	98.9	85.9
positive predictive value (%)		96.6	99.1	91.3
negative predictive value (%)		95.9	100	94.7

**Table 4:** Discrimination of samples positive for HAV due to vaccination or natural infection.

HAV Status	Number of Reactive Antigens			
	0	1	2	3
vaccination (*n* = 15)	9 (60%)	5 (33.3%)	1 (6.7%)	0 (0%)
natural infection (*n* = 20)	0 (0%)	0 (0%)	1 (5%)	19 (95%)

**Table 5:** Identification of samples with and without vaccination against hepatitis B virus (HBV).

		HBsAg ad	HBsAg ay
classification	true positive	29	26
	false positive	1	0
	false negative	1	4
	true negative	14	15
diagnostic accuracy (%)		95.6	91.1
sensitivity (%)		96.7	86.7
specificity (%)		93.3	100
positive predictive value (%)		96.7	100
negative predictive value (%)		93.3	78.9

**Table 6:** Results of the influenza vaccination study.

Pathogen	Classification	Study Participants	
		*n* = 34	%
**HAV**	negative	5	14.7
	positive	25	73.5
	ambiguous	4	11.8
	positive by vaccination	21	84.0
	positive by infection	4	16.0
**HBV**	negative	29	85.3
	positive	3	8.8
	ambiguous	2	5.9
	positive and cleared	2	66.7
	positive and non-cleared	1	33.3
	vaccination	12	41.4
**HCV**	negative	28	82.4
	positive	0	0
	ambiguous	6	17.6
	acute infection	0	0
	chronic infection	0	0
**CMV**	negative	7	20.6
	positive	24	70.6
	ambiguous	3	8.8
***T. gondii***	negative	10	29.4
	positive	23	67.6
	ambiguous	1	2.9
***H. pylori***	negative	20	58.8
	positive	11	32.4
	ambiguous	3	8.8

**Table 7:** Classification results and signal-to-cutoff (S/CO) values of two study participants ambiguous for HCV.

				S/CO Value				
Study Participant	Days after Influenza Vaccination	Classification	Acute or Chronic	Core g4a	Core g1b	c22 g1a	NS3 g1a	NS3 g1b
1	0	negative	-	0.0	0.1	0.1	0.0	0.3
	3	negative	-	0.0	0.1	0.3	0.0	0.6
	7	positive	acute	0.0	0.1	0.0	65.6	6.8
	21	positive	acute	0.0	0.2	0.1	313.8	23.2
	70	positive	acute	0.0	0.1	0.1	43.5	4.4
2	0	positive	acute	0.0	0.4	1.7	104.2	3.5
	3	positive	acute	0.7	0.5	1.6	121.5	4.0
	7	positive	acute	1.5	0.4	1.3	92.8	3.2
	21	positive	acute	0.1	0.3	1.0	68.6	2.5
	70	borderline	-	0.7	0.2	0.9	53.7	2.0
